# Simulating dynamic facial expressions of pain from visuo-haptic interactions with a robotic patient

**DOI:** 10.1038/s41598-022-08115-1

**Published:** 2022-03-10

**Authors:** Yongxuan Tan, Sibylle Rérolle, Thilina Dulantha Lalitharatne, Nejra van Zalk, Rachael E. Jack, Thrishantha Nanayakkara

**Affiliations:** 1 Dyson School of Design Engineering, Imperial College London, London, SW7 1AL UK; 2 School of Psychology & Neuroscience, University of Glasgow, Glasgow, G12 8QB UK

**Keywords:** Biomedical engineering, Physical examination, Mechanical engineering, Human behaviour

## Abstract

Medical training simulators can provide a safe and controlled environment for medical students to practice their physical examination skills. An important source of information for physicians is the visual feedback of involuntary pain facial expressions in response to physical palpation on an affected area of a patient. However, most existing robotic medical training simulators that can capture physical examination behaviours in real-time cannot display facial expressions and comprise a limited range of patient identities in terms of ethnicity and gender. Together, these limitations restrict the utility of medical training simulators because they do not provide medical students with a representative sample of pain facial expressions and face identities, which could result in biased practices. Further, these limitations restrict the utility of such medical simulators to detect and correct early signs of bias in medical training. Here, for the first time, we present a robotic system that can simulate facial expressions of pain in response to palpations, displayed on a range of patient face identities. We use the unique approach of modelling dynamic pain facial expressions using a data-driven perception-based psychophysical method combined with the visuo-haptic inputs of users performing palpations on a robot medical simulator. Specifically, participants performed palpation actions on the abdomen phantom of a simulated patient, which triggered the real-time display of six pain-related facial Action Units (AUs) on a robotic face (MorphFace), each controlled by two pseudo randomly generated transient parameters: rate of change $\beta$ and activation delay $\tau$. Participants then rated the appropriateness of the facial expression displayed in response to their palpations on a 4-point scale from “strongly disagree” to “strongly agree”. Each participant ($n=16$, 4 Asian females, 4 Asian males, 4 White females and 4 White males) performed 200 palpation trials on 4 patient identities (Black female, Black male, White female and White male) simulated using MorphFace. Results showed facial expressions rated as most appropriate by all participants comprise a higher rate of change and shorter delay from upper face AUs (around the eyes) to those in the lower face (around the mouth). In contrast, we found that transient parameter values of most appropriate-rated pain facial expressions, palpation forces, and delays between palpation actions varied across participant-simulated patient pairs according to gender and ethnicity. These findings suggest that gender and ethnicity biases affect palpation strategies and the perception of pain facial expressions displayed on MorphFace. We anticipate that our approach will be used to generate physical examination models with diverse patient demographics to reduce erroneous judgments in medical students, and provide focused training to address these errors.

## Introduction

Simulation based education offers safe, controlled, and effective learning environments^1^ for medical students to practice hands-on physical examination skills. They can explore different manoeuvres on physical mannequins or tissue phantoms in their own time after bed-side teaching to facilitate self-learning and increase teaching and training efficiency^2^. However, these systems have different levels of fidelity. For example, the highest level of fidelity are standardised patients (SPs) who are professionally trained actors simulating patient behavior such that skilled clinicians cannot detect the simulation. Although the use of SPs can improve patient outcomes^3^, SP training and skill maintenance are time-consuming, and a diverse SP pool must be maintained to achieve various medical examination learning objectives and cultural competence^4^. Robotic medical simulator such as physical mannequins offer lower fidelity than SPs, but can simulate a greater variety of medical conditions and respond to physical inputs with movement, haptic, visual and auditory feedback that enables students to practice more specific procedures^5^.

In both SPs and physical mannequins, simulating patients with different demographic identities such as age, gender, and ethnicity is challenging^6^. Some commercially available mannequins such as the Paediatric HAL^7^ offer gender and skin colour variations, but options are limited and cannot easily be swapped between training sessions. In contrast, virtual simulators such as Virtual Patients^8^ can render a wide variety of patients with different demographics, medical history, and clinical scenarios. However, interactions are often only verbal or dialogue-based and they do not respond to physical actions from users. Physical-virtual systems such as the Virtual Breast Exam Patient^9^ can simulate multiple patient identities and respond to physical inputs such as touch via visual feedback such as facial expressions at a higher level of detail than physical mannequins using mixed reality or projections. Together, these features could substantially enhance the training experience by allowing physicians interacting with phantoms and mannequins which resemble human patients.

Facial expressions are frequently used to communicate one’s internal states to others and are crucial in medical consultations where patients provide information using verbal and non-verbal means^10,11^. Pain is commonly exhibited during medical examinations and often communicated through facial expressions^12^. Therefore, if physicians wrongly interpret the severity of the patients’ pain from their facial expressions, patient discomfort can be ignored, which can increase the risk of mistreatment or even mortality^12,13^. For example, certain demographic factors such as patient gender and ethnicity can impact the interpretation of facial expressions of pain, which can negatively impact the physician-patient relationship and trust^14–16^. Therefore it is crucial for physicians to correctly interpret the patient’s pain in clinical diagnoses.

Multiple virtual platforms and systems exist that can generate human avatars and dynamic facial expressions with fine details. FaceGen Modeller^17^ uses photos of real people to manually generate 3D meshes of their faces. The more recently released MetaHuman^18^ enables mass scale generation of human avatars with detailed facial and bodily features and clothing. Our recently developed robotic face MorphFace^19^, which is based on the MakeHuman^20^ and FACSHuman^21^ systems, can simulate six human avatars (females and males of Asian, Black and White ethnicity), can be integrated with data capturing devices such as the force sensor to render real-time facial expressions in response to palpation forces, making it a versatile physical-virtual simulation tool.

Recent studies on the perception of pain experience show systematic biases among the general public and medical providers^22^. For example, individuals with lower socioeconomic status are believed to be less sensitive to pain regardless of their ethnicity or gender^22^. Similarly, pain and other negative facial expressions such as sadness are more readily detected on White than Black male faces^23^. Together, these studies outline robust biases in the perception of pain across groups, highlighting the need to expose medical students to patients with different demographic and social backgrounds, and implement mechanisms to identify and reduce racial and social bias in treatment^24,25^.

**Figure 1:**
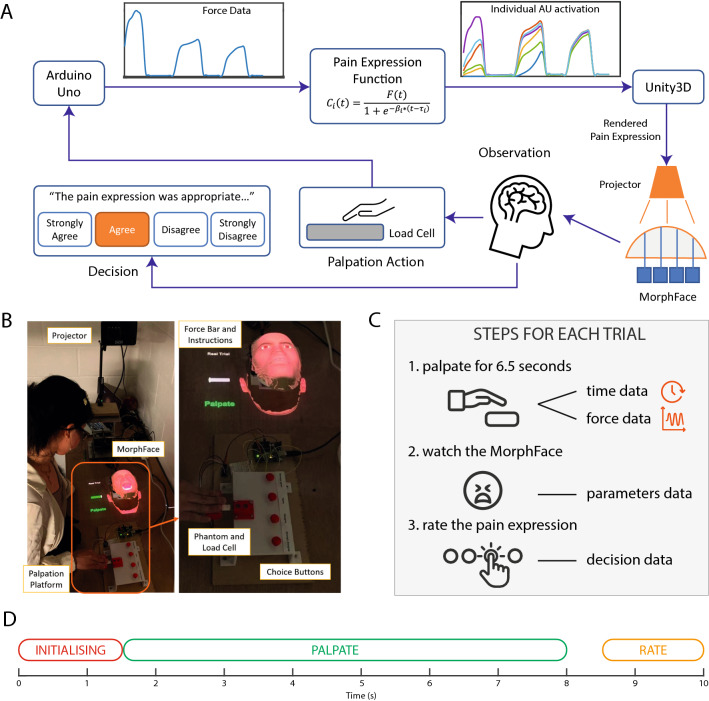
(**A**) Schematics of the interactive platform. (**B**) Experiment setup. (**C**) Experiment plan. (**D**) Trial sequence.

Thus a training platform that can present pain-related expressions of patients from various demographic backgrounds is required. Here, we addressed this critical limitation by developing an interaction platform that can evaluate the perceptual impact of transient dynamics of pain facial expression using MorphFace based on participant responses for diverse patient groups. Specifically, we used a sensorised silicon phantom to interface the force captured during the abdominal palpation procedure—a common practice in physical examination—to dynamically modulate the pain facial expressions displayed by a demographically variable set of MorphFace human avatars. An overview of the MorphFace system and task procedure is shown in Fig. 1A^26^. Using this system, we conducted a data-driven behavioural experiment to find the transient temporal facial movement activation parameters that simulate dynamic pain facial expressions that participants perceived to be appropriate through multiple palpation and observation trials, as shown in Fig. 1C. Our approach differs from previous approaches to studying facial expressions of pain in that participants induced simulated pain to the patient through their actions, as shown in Fig. 1B. For example, prior work modelling facial expressions for pain across cultures^27^ asked users to view randomly generated dynamic facial expressions displayed on a computer screen and to indicate which facial expressions accurately represented the broad category of pain. Here, we refine this paradigm by asking participants to also perform actions (abdominal palpation) that would directly affect the exhibition of such facial expressions, thus enabling the modelling of facial expressions of pain exhibited within a given medical context and specific user interactions: pain received by the patient and subsequently the facial expressions of pain arise from palpation of the abdomen performed by the participant. Thus, we anticipate that our system and procedure can be used to create dynamic pain facial expression simulation models of interactive physical examination procedures for diverse patient identities, ultimately providing bespoke medical training solutions to reduce erroneous judgments in individual medical students due to perceptual biases.

## Results

We instructed participants (detailed information can be found in “Experiment protocol with human participants”) to palpate on a silicon phantom (simulating a painful area of the lower abdomen), observe pain facial expressions exhibited on MorphFace, and rate its appropriateness (“strongly agree”, “agree”, “disagree” or “strongly disagree” that the expression was appropriate) given the force applied within a time limit of 6.5 s. Pain facial expressions changed in real-time with palpation forces captured by the phantom. Multiple palpation actions could be performed during each trial.

We controlled the generation of the facial expressions using two randomly generated transient parameters for each AU-the rate of change $\beta$ and delay $\tau$. For each trial we captured the force profile, the randomly generated parameters, and the participant rating. Recording the force profiles enabled measurement of the variability in behaviours across participants and MorphFace. The parameter values and corresponding ratings enabled us to find parameter ranges that best simulate the pain facial expression from palpation actions.

**Figure 2:**
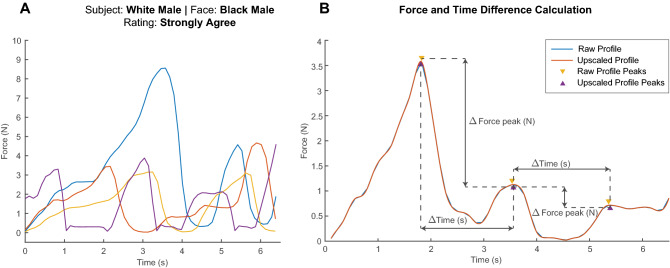
(**A**) Force profiles recorded from four trials of a White male participant palpating a Black male MorphFace patient who “strongly agreed” that the pain facial expression displayed was appropriate given his palpation actions. (**B**) Force and time differences between successive palpation actions were based on the peaks of the upsampled force profiles.

Figure 2A shows the recorded palpation force profiles from four trials performed by a White male participant viewing a Black male MorphFace on trials where they rated the pain expressions as “strongly agree” (i.e., most appropriate given the palpation actions applied). Each line in Fig. 2A shows a different number of peaks and different force levels at each peak, suggesting that the participant varied his palpation strategy across trials even though the demography of the MorphFace and his rating remained the same.

The time differences between the force peaks represent the time the participant took to view the facial expression and plan the next palpation action. Differences in force peak magnitude show the force variations the participant applied to the phantom. Together they represent the palpation behaviour of the participant. Each participant performed 50 trials on each of the 4 MorphFace identities (shown in Fig. S1) shown in random order of the demography of MorphFace for each participant. To better identify the force peaks, we fitted cubic splines to upsample the raw force data using methods detailed in “Force data upsampling”. Figure 2B shows the comparison between raw and upsampled data for one force profile. The error between the force peaks in the upsampled and those in the raw data for all trials have an error distribution of $0.025 \pm 0.0025$ N, which is negligible compared to the mean peak force of 3.10N.

**Figure 3:**
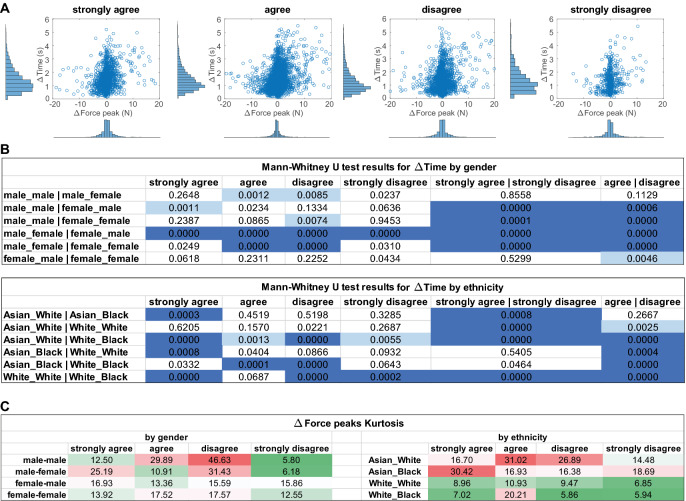
(**A**) Distributions of the change in force and time between two consecutive palpation force peaks for all trials grouped by participant ratings. (**B**) Mann-Whitney U test results of time differences ($\delta t_p$) between interaction groups. For gender-wise comparisons, “male_male | male_female” denotes $\delta t_p$ difference between male participants examining the male MorphFace and male participants examining the female MorphFace. Same format applies to ethnicity-based comparisons. Light blue represents $p < 0.01$ and dark blue represents $p < 0.001$. (**C**) Kurtosis of force variations ($\delta f_p$) between interaction groups. Higher kurtosis of $\delta f_p$ means a sharper peak around $f_p = 0$.

To examine the potential effects of demographic differences on palpation behaviour between participant-MorphFace pairs, we computed the joint distribution of $\delta t_p = t_p(i) - t_p{(i-1)}$ and $\delta f_p = f_p(i) - f_p(i-1)$, where $t_p(i)$ is the time at the *i*th force peak, and $f_p(i)$ is the *i*th force peak. Figure 3A shows the force and time differences between palpation actions within and across all trials grouped by ratings. See Supplementary Fig. S3 for details of all gender interactions and Fig. S4 for all ethnicity interactions. Gender interaction analysis included all ethnicity data and vice versa for ethnicity interaction analysis. Figure 3B shows the *p* values of Mann-Whitney U test which compares $\delta t_p$ between different participant-MorphFace interaction pairs. Figure 3C shows the kurtosis of $\delta f_p$ which shows the force variations between the interaction pairs.

The $\delta t_p$ is consistently statistically significantly different ($p < 0.001$) between male-female (male participants interacting with female MorphFace patients) vs. female-male examination across all four response options. $\delta t_p$ for male participants was less positively skewed than for female participants, suggesting that male participants might spend more time viewing the facial expression and/or planning for the next palpation actions. In opposite gender interactions, the variation of the size of the peak force $\delta f_p$ was similar (ratio of kurtosis $< 1.5$) for “agree” or “strongly agree” cases. However, male participants viewing female MorphFace identities showed noticeably higher concentration of $\delta f_p$ around zero (ratio of kurtosis $> 1.5$) for the “disagree” case, while female participants viewing male MorphFace identities showed the same pattern for the “strongly disagree” case. This suggests that participants tended to apply the next palpation action more quickly when they disagree with appropriateness of the facial response for opposite gender interactions. For same gender interactions between the participant and MorphFace, males and females were significantly different in $\delta t_p$ variation only for the “disagree” case ($p < 0.01$). In terms of the variation of $\delta f_p$, male-male had higher ratio of kurtosis compared to female-female (ratio of kurtosis $> 1.5$) for “agree” or “disagree” cases. Thus, for same gender interactions, male participants made relatively smaller increments and decrements in their peak forces to closely observe the corresponding pain facial expression changes when decisions were not apparent.

We found no significant differences in $\delta t_p$ between Asian-White (Asian participants interacting with White MorphFace) vs. White-White regardless of the decision cases. $\delta t_p$ of Asian-White vs Asian-Black showed significant difference in the “strongly agree” case, and $\delta t_p$ of White-White vs. White-Black showed significant differences in all cases except for “agree”. This suggests Asian participants spend a similar amount of time examining White and Black MorphFace except when the rating is “strongly agree”, whereas White participants spent a different amount of time examining White and Black MorphFaces except for the “agree” case. This could be due to less familiarity with White and Black faces amongst Asian participants, whereas White participants had interactions with their own-ethnicity faces in which they may have had perceptual expertise of^28^, resulting in the significant $\delta t_p$ differences between the same (White-White) and different (White-Black) ethnicity interaction cases. Trials performed by Asian participants (8 participants, 1545 trials) showed a higher concentration of $\delta f_p$ around zero than White participants (8 participants, 1550 trials) in most of the cases, reflecting that they varied the palpation forces less frequently than White participants. White participants showed similar variations in $\delta f_p$ when interacting with White and Black MorphFace, though in the “agree” case they varied their force less when viewing Black compared to White MorphFace patient faces.

**Figure 4:**
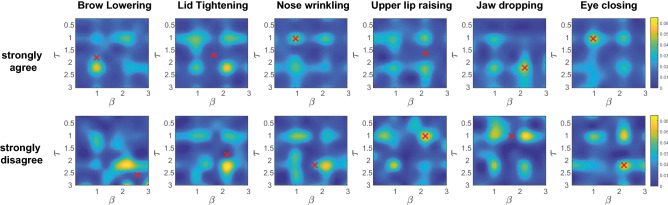
Transient parameter probability distributions between the two extreme rating cases (“strongly agree” and “strongly disagree”) for each AU across all trials. The two transient parameter values are randomly generated (see “Experimental setup” for detail) for each AU in each trial. High probability in the parameter values represent the AU activations of generated facial expression that all participants rated as “strongly agree” or “strongly disagree” for appropriateness. Clusters of peak joint probability are accompanied by branches along one parameter of the other, implying considerable joint and marginal probability distributions. To account for both patterns, we used the absolute peak of the entire probability landscape as well as the peak of the marginal probability distribution (by taking the sum of the probability landscape along one parameter axis to identify the peak corresponding to the other parameter). This resulted in a weighted average based on the relative values of two probability peaks (the absolute peak and the peak of the marginal distribution). This was done for both parameters. The red cross indicates the coordinates of the weighted averages.

Next, we examined what transient parameter values of the pain facial expressions displayed on MorphFace that participants rated as “strongly agree” and “strongly disagree” for appropriateness. Figure 4 shows the probability distribution of ratings of each parameter pair: rate of change $\beta$ and delay $\tau$, calculated using the method from “Probability distribution of transient parameters”. For “strongly agree”, we observed low rates of change $\beta < 2$ for all AUs except Upper Lip Raiser (AU10) and Jaw Drop (AU26). Jaw Drop (AU26) also had longer delays $\tau > 2$ compared to other AUs, with the shortest delay associated with Eyes Closed (AU43) $\tau \approx 1$.

**Figure 5:**
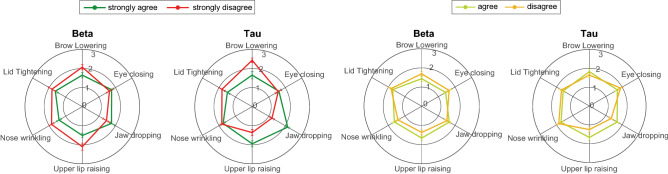
Parameter variations ($\beta$ and $\tau$) for different AUs between decision cases. The error bars along the polar axes represent the standard error across participants.

Figure 5 shows the probability distribution of the weighted average parameter values (centroids) grouped by AUs for all ratings by all participants. In the “strongly agree” case, the rate of change are $1.26< \beta < 1.50$ for AU4 (Brow Lowerer), AU7 (Lid Tightener), AU9 (Nose Wrinkler), and AU10 (Upper Lip Raiser), but $1.56< \beta < 1.96$ for AU43 (Eyes Closed) and AU26 (Jaw Drop), suggesting the activation intensity of AU43 and AU26 increase more rapidly with the applied palpation force than other AUs. The delays are $1.34<\tau < 1.86$ for AU4 and AU7 and $1.78<\tau < 2.25$ for AU26 and AU10, with the latter two AUs having a longer delay than the former two. For AU4 and AU26, we found significant differences in the $\tau$ parameters (Mann-Whitney U-test, $p<0.01$) between “strongly agree” and “strongly disagree” cases. For the “agree” and “disagree” cases, we found no significant differences between the parameters for any of the AUs. When combining “strongly agree” with “agree”, and “strongly disagree” with “disagree”, we found significant differences between the $\tau$ value for AU26 (Mann-Whitney U-test, $p<0.01$). This shows that the activation delay of AU26 (Jaw Drop) is significantly associated with participant ratings of appropriateness of the displayed pain facial expressions.

**Figure 6:**
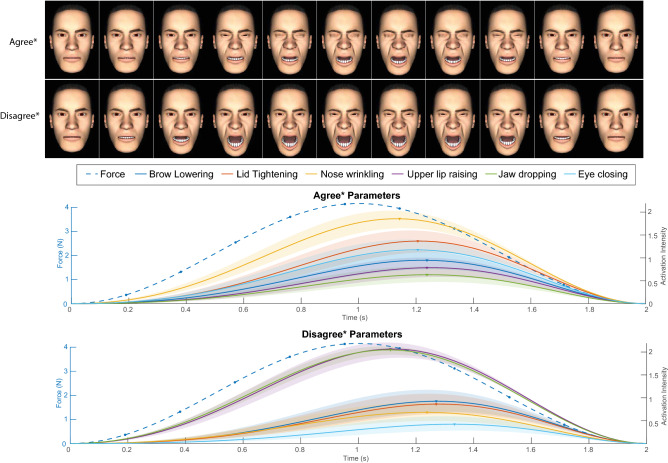
Simulated results using median parameters of all trials rated “strongly agree” and “agree”, marked as “agree*”; and “disagree” and “strongly disagree”, marked as “disagree*”. The shaded regions represent standard errors from individual participant ratings.

Figure 6^29^ shows the simulated pain facial expression generated using the median transient parameters from trials rated “strongly agree” and “agree” (“agree*”), and those rated “strongly disagree” and “disagree” (“disagree*”) from all participants. Activation intensities of the AUs are plotted with a simulated sine wave force profile following the method from “Simulation Using transient parameter pairs”, the shaded regions represent standard error across participants. The activation intensity patterns for “agree*” are more synchronous than for “disagree*”.

## Conclusions

We modelled dynamic facial expressions of pain using a data-driven perception-based psychophysical method combined with visuo-haptic interactions of users applying palpation examinations to a robotic medical simulator. We controlled the dynamic response of six pain-related AUs using two transient parameters—the rate of change ($\beta$) and delay ($\tau$)—to render pain facial expressions on four face identities of different gender and ethnicity demographics. We found that the activation delay of AU26 (Jaw Drop) significantly influenced the perceived appropriateness of the pain facial expression of the simulated face undergoing abdominal palpation such that a longer delay was viewed to be more appropriate. Moreover, a gradual decrease of intensity and speed of response from upper face AUs (around the eyes) to those in the lower face (around the mouth) is a common feature of facial expressions rated as appropriate by all participants. Analysis across different demographic interactions between the participants and the Morphface showed differences in palpation behaviors including the duration between force peaks, controlling the size of consecutive force peaks, and the corresponding transient parameters of the facial expressions rated as appropriate. As gender and ethnicity are the only two dominant factors that varied across participants and in the Morphface, our results suggest that gender and ethnicity interactions underpin this variance. These findings highlight the usefulness of visuo-haptic interactions with a robotic patient as a method to quantify differences in behavioral variables relating to medical examination with diverse participant groups and to assess the efficacy of future bespoke interventions aimed at mitigating the effects of such differences.

## Discussion

Participants showed different palpation behaviours based on the perceived appropriateness of the pain facial expressions. Figure 3A as well as Supplementary Figs. S3 and S4 show that when participants rated “strongly disagree”, they tended to vary $\delta f_p$ in a small range (Kurtosis = 11.66). In the cases of “agree” and “disagree” the $\delta f_p$ kurtosis are high (19.75 and 21.46), showing that participants varied their palpation force in a broader range. Similarly we observed statistically significant differences in $\delta t_p$ between the rating “strongly agree” and “strongly disagree” in three gender-pair and four ethnicity-pair comparison cases as shown in Fig. 3B. Such variations in palpation behaviours could have assisted in decision-making (i.e., rating the appropriateness of the facial expression) by optimising the balance between effort (i.e., number of palpations applied and force variation) and information gain for perceptual decision.

The probability distribution of the AU transient parameter pairs rated as “strongly agree” in Fig. 4 shows multiple oriented local high joint probability distributions in $\beta$ and $\tau$. The orientations often tend to be parallel to one of the parameter axes, suggesting local regions where the effect of one parameter is stronger than the other. The pattern of these oriented distributions is reminiscent of a lattice shape, highlighting the inter-connectivity between the two parameter pairs, as the participants only perceived the resultant visual output where certain combinations of the two parameter values may render similar facial expressions. See Supplementary Figs. S5–S8 for detailed results across all gender and ethnicity interaction contexts. This suggests that in general, the four combinations of low/high delay ($\tau$) and low/high rate of change ($\beta$) triggers the perception of the appropriateness of the facial expression given palpation forces. However, it does not indicate the specific combinations across the six AUs. Therefore, we used a weighted average to compute a single centroid as explained in Fig. 4 caption.

Differences in the weighted averages of $\beta$ and $\tau$ values are larger between “strongly agree” and “strongly disagree” than between “agree” and “disagree” cases as shown in Fig. 5. AUs with larger movements such as AU26 (Jaw Drop) varied most $\tau$ between “strongly agree” and “strongly disagree” cases with a larger $\tau$ associated with more appropriate ratings, suggesting the AU should be activated later in the facial expression. In contrast, AUs with smaller movements such as AU4 (Brow Lowerer) and AU7 (Lid Tightener) are perceived as more appropriate when the $\tau$ value is small (i.e., shorter delay), suggesting that the AU should be activated earlier in the facial expression. The variation of $\beta$ across all AUs for the “strongly agree” case is smaller than for the “strongly disagree” case, and higher $\beta$ in AUs with large movement were shown to be more appropriate. We speculate that these activation patterns could be related to the volume of the corresponding facial muscle groups and the metabolic costs of the muscle contraction, where low $\tau$ groups could have lower metabolic costs than high $\tau$ groups.

We observed a variety of different effects associated with demographic factors regarding preferences for the weighted averages of transient parameters and comparing all rating cases. Results for gender are shown in Supplementary Fig. S9 and those for ethnicity are shown in Supplementary Fig. S10. We discuss a few key AUs here for brevity. Specifically, we found a slower rate of eye closing (AU43) accompanied by a shorter delay in participant response across all gender interactions. Participants perceived faster facial responses to be more appropriate for White MorphFace when viewed by both ethnic groups of participants. Similarly, for White MorphFaces, White participants perceived shorter delay facial expressions as most appropriate whereas Asian participants perceived longer delays to be more appropriate. Examination of the effect of individual AUs such as AU7 (Lid Tightener), both genders perceived as most appropriate a longer delay of AU7 (Lid Tightener) on male faces and faster activation of AU7 on female faces, whereas male participants vs. male MorphFace perceived shorter delays as most appropriate and female vs. female perceived longer delays as most appropriate; Asian participants perceived slower rate of change as most appropriate, and White participants perceived shorter delays as most appropriate while Asian participants perceived longer delay as most appropriate. These findings suggest that bespoke facial pain expression models could be derived for different gender-ethnicity interaction scenarios with a focus on exposing and reducing perception biases.

The activation intensities of AUs in Fig. 6 shows that compared to the parameters with “disagree*”, the “agree*” parameters have higher synchrony. At maximum activation intensities, the AUs used to generate pain facial expressions that participants rated as “agree*” (i.e., most appropriate) are ordered relative to the facial muscle sizes and visual changes in displacement, which is consistent with our speculations based on our analysis of the weighted averages of individual AUs. Specifically, AUs with smaller muscle volumes such as AU9 (Nose Wrinkler), AU7 (Lid Tightener), and AU4 (Brow Lowerer) activate more quickly and intensely than AUs with larger muscle volumes such as AU26 (Jaw Drop) and AU10 (Upper Lip Raiser). This is corroborated by observing that trials rated as “disagree*” comprise activation of AU26 and AU10 earlier and more intensely than the other AUs.

In this paper, the general focus is to derive a model of the best transient AU parameters that is valid across known groups of people using a novel robotic patient framework. The current study included participants of White and Asian ethnicity, in the same age group and sex-matched. However, our sample did not include other ethnicities including Black participants, who might represent pain facial expressions differently than other groups. Troy highlighted the needs to consider both ethnic and cultural influences^30^, and even our limited diversity in ethnicity in this paper showed that pain expressions driven by dynamic parameters depended on the sex and ethnicity interaction, and further studies will be done to test if this holds for broader classes of interaction, which is currently beyond the scope of this paper. Therefore, we invite caution in attributing the generalizability of our results across ethnicities and highlight the need to conduct further studies that include Black participants. A library containing pain expression models derived via homo and hetero interactions by sex and ethnicity will help us to better understand the visual stimuli of biases in recognising and expressing pains, in attempt to reduce biases in pain expression recognition in primary medical examinations via quantitative analysis of the expressions.

Our findings demonstrate homogeneity in the AU transient parameter values associated with pain facial expressions in response to palpation force in this visuo-haptic task, and diversity according to gender and ethnic patient-participant interactions. Participants generally agreed that appropriate pain facial expressions for low intensity pain comprise certain early onset upper face AUs, which could relate to their lower muscle volume and/or visual salience. In contrast, when the induced pain intensity is high, pain facial expression comprise more lower face AUs that could have larger muscle volume and involve higher metabolic costs. We also found gender and ethnicity differences in the temporal regulation of force peaks. However, in general, participants increased the variation of the range of force peaks when their conviction about the decision is low (agree or disagree) compared to when their conviction was high (strongly agree or strongly disagree). This was accompanied by a higher positive skewness to use smaller temporal gaps $\delta t_p$ between force peaks. This pattern could reflect a visual information gain process whereby lower conviction of the outcome reflected from the visual information leads to larger variation in force peaks with shorter temporal gaps.

## Methods

### Experimental setup

We developed a novel interactive facial expression evaluation platform to generate and evaluate a set of temporal facial movement parameters for synthesising pain facial expressions that users find appropriate for simulated patients undergoing abdominal palpation, as shown in Fig. 1A. Participants performed palpation actions on a block of silicon (Ecoflex 00-10, Smooth-On, Inc, USA) abdomen phantom placed on top of a load cell (20KG Weight Sensor with HX711 Amplifier, DIYmalls). The applied palpation force is captured by the load cell and sent to Unity3D (Unity Technologies, USA) via an Arduino Uno through a serial port at a baud rate of 9600. A pain expression generation function is implemented in C# in Unity3D that describes the following relationship between palpation force and the AU activation intensity:

1 $$\begin{aligned} C_i(t)= \frac{F(t)}{1+e^{-\beta _i (t-\tau _i)}}, \; ~\mathrm{for} \; i=4,7,9,10,26,43 \end{aligned}$$

where $C_i(t)$ is the activation intensity of the *i*th facial activation unit (AU) at time *t*. *F*(*t*) is the palpation force at time *t*. $\beta _i$ defines the gradient of the increasing sigmoid function for the *i*th AU (rate of change), and $\tau _i$ is the activation delay of the *i*th AU.

We included six Action Units (AU4: Brow Lowerer, AU7: Lid Tightener, AU9: Nose Wrinkler, AU10: Upper Lip Raiser, AU26: Jaw Drop and AU43: Eyes Closed)^31^ as these have been shown to be present in models of pain expressions of different intensities and across different cultures^6,27^. Using MakeHuman^20^ with the FACSHuman^21^ plugin, we generated a natural expression mesh and 6 maximum AU activation mesh for each AU. We constrained the maximum activation intensities $max(C_i)$ by the default mesh deformation defined in the FACSHuman plugin. We imported these meshes into Blender, assigned a $shape key = 1.0$ to each AU with $max(C_i)$, and added to the natural expression which had $shape key = 0$. The shape key values represented the activation intensities for the AUs and could be controlled individually. We separated the head of the human avatar from the body and exported to Unity3D, where the corresponding mesh deformation for each AU could be controlled by setting their shape key values. Supplementary Figure S2 shows the natural expression and maximum pain expression synthesised using these AUs.

### Experiment protocol with human participants

We used the abdominal palpation platform shown in Fig. 1 to find what $\beta _i$ and $\tau _i$ should be assigned to the pain facial expression generating function to render appropriate pain facial expressions for a simulated patient undergoing abdominal palpation. For each AU, the rate of rise $\beta _i$ and delay of response $\tau _i$ were assigned a random number between 0 and 3 at increments of 0.3, the range and increment were both chosen based on results from a small pilot study on 4 volunteers. We projected a dynamic bar plot next to the face as visual feedback for participants to regulate their palpation force. The maximum value represented by the bar was 3N as the palpation force applied on the abdomen had been shown to be between 0.09*N* and 2.22*N*^32^. We created four face identities comprising two genders (male and female) for each of two ethnicity (Black and White). Supplementary Figure S1 shows these four face identities rendered on MorphFace.

We recruited undergraduate students from Imperial College London (n = 16, 4 Asian females, 4 Asian males, 4 White females, and 4 White males, aged 18 to 23 years (M = 20.9 years, SD = 2.14 years)) to participate in this experiment. Participants provided informed consent to take part in the experiment.

The study was approved by the Imperial College Research Ethics Committee (no. 20IC6295). All methods were carried out in accordance with the approved protocol and comply with general guidelines.

We briefed participants on the experimental protocol and asked them to read the experiment instruction document on arrival. We advised participants verbally that the MorphFace and the silicon phantom represents a patient with an underlying physiological condition that causes pain when the silicon phantom is pressed. We instructed participants to palpate the silicon phantom and observe the facial expressions projected onto MorphFace for each of 4 different faces with 50 trials per face identity. The appearance of the faces is shown in Supplementary Fig. S1. After viewing the facial expression, participants were tasked to respond to the statement “The facial expression is appropriate given the palpation force you applied” by pressing one of four buttons representing a 4-point Likert scale ranging from “strongly agree”, “agree”, “disagree” to “strongly disagree”. This statement was given verbally and was printed in bold on the experiment instruction document which the participants could view during the experiment. The physical setup and the interactive pad are shown in Fig. 1A. We used two laptops to record force data, compute and render facial responses, and record responses (Intel Core i7 2.5GHz, 16 GB memory, AMD Radeon R9 M370X), both using the same compiled Unity3D 2019.3.7f1 executable. We saved all randomly generated parameters, palpation forces, and participant responses as text files at the end of each trial inside the compiled executable.

The stages for each trial are summarised below and shown in Fig. 1D. We included the same text in the experiment instruction document:

1. “Initialising”: This text is highlighted in red and projected onto the tabletop next to the face. We instructed participants not to touch the phantom during this stage.
2. “Palpate”: This text is highlighted in green and projected next to the face. A force bar with green filled colour appeared below the text, indicating that the participant can now perform palpation on the phantom. The time limit of 6.5 s is not shown in the projection.
3. “Rate”: This text is highlighted in yellow and projected next to the face. We instructed participants to press one of the four buttons to register their response to the appropriateness of the facial expression displayed.
4. “Get Ready”: This text is highlighted in white and projected next to the face. Another text string projected above this text box showed the current trial number and total number of trials.

Participants performed practice trials using an Asian male face (not used in the experiment) until they felt confident to perform the set of actions described above.

### Exclusion criteria

**Figure 7:**
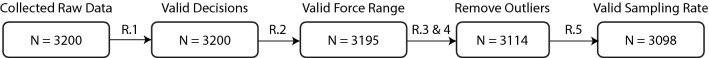
Exclusion criteria on collected trial data. R.X represents an exclusion rule.

We collected a total of 3200 data points (16 participants, 50 trials per MorphFace identity, 4 identities). Although the experimental protocol and plan had been explained to the participants, mistakes during the experiment were made. We therefore introduced the following exclusion criteria to remove invalid trials (as shown in Fig. 7):

1. Decision is not empty—participant made a choice and the choice was registered for that trial.
2. The minimum force for a trial is greater than 0—participant did not touch the silicon phantom during the ’Initialising’ stage.
3. The number of peaks (palpation actions) is greater than 0—participant varied the force during the trial.
4. The number of peaks is less or equal to the upper adjacent value (UAV) calculated from filtered results from the previous three criteria (UAV = 9).
5. The number of force samples collected for a trial is greater than 2 times of the UAV (18).

3098 were labeled to be valid with an exclusion rate of $3.19\%$.

### Data analysis

#### Force data upsampling

We only considered the peaks of palpation force for analysis. We obtained force peaks using *findpeaks* function in MATLAB (version 2019b, Mathworks Inc, Natic, MA, USA) with minimum prominence greater than 0.1 after upsampling force data by a factor of 5 using cubic spline fitting (*spline* function in MATLAB). The median number of force peaks were 3 with an upper adjacent value of 9 within the 6.5 s sampling window. Therefore trials with less than 18 samples out of the two computers were excluded from the analysis, and the remaining trials are above the minimum required sampling frequency.

The same peak detection function was applied on the upsampled force data to extract the magnitude and timestamp of the peaks for each palpation cycle. The differences in peak forces and the timestamps were calculated and grouped by participant gender and ethnicity, as shown in Figs. S3 and S4.

The time differences for the first and last 10% of trials done by the participants were compared to see if the participants had enough practice such that they did not perform differently over the real trials. A Mann-Whitney U test was performed and returned $p=0.41$ such that there was no statistically differences between the time differences in the first and last 10% of the trials, meaning they did not spend more or less time between their palpation actions between the start and the end of the experiment.

#### Probability distribution of transient parameters

The randomly generated transient parameters $\beta$ and $\tau$ were grouped based on the corresponding ratings of trials. The probability distribution of the ranges of $\beta$ and $\tau$ were calculated by dividing their appearance frequencies by the total frequencies after they were placed in bins with a size of 0.4. The weighted averages of the probability distribution were calculated as a weighted average of the absolute peak and the peak of the marginal (row/column wise) distribution. Supplementary Figures S5–S8 show the probability distribution of these transient parameters grouped by gender and ethnicity of the participants.

#### Simulation using transient parameter pairs

We grouped and calculated the median of the weighted averages of the transient parameter pairs for all trials rated with “strongly agree” and “agree”, and “disagree” and “strongly disagree” to simulate facial expressions using a sine wave as the force profile with an average of 2N and a period of 2 s. Video clips by ethnicity and gender pairs can be viewed at simulation video clips.

## Supplementary Information

Supplementary Information.

## Data Availability

The datasets generated and analysed during the current study are available in the OSF repository.
