# Supplementary material for: Simulating dynamic facial expressions of pain from visuo-haptic interactions with a robotic patient
Source: Sci Rep. 2022 Mar 10;12:4200. doi: 10.1038/s41598-022-08115-1 (PMC8913843; doi:10.1038/s41598-022-08115-1)
Supplement: Supplementary file 1 — Supplementary Information. [file 41598_2022_8115_MOESM1_ESM.pdf]

Supplementary Material

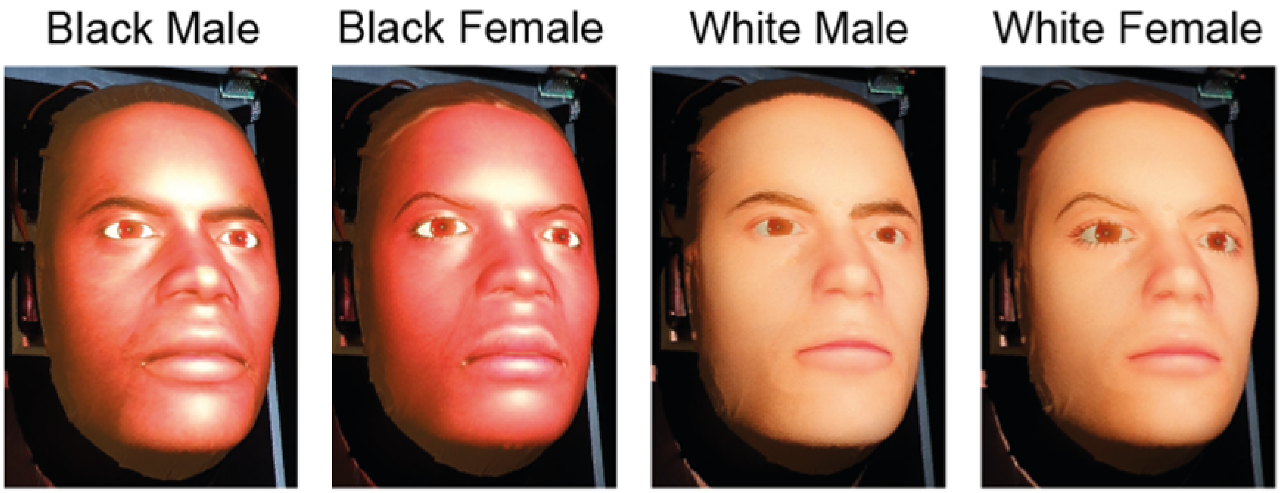

Figure S1. Photos of the four face identities (Black male, Black female, White male, White female) rendered in MorphFace

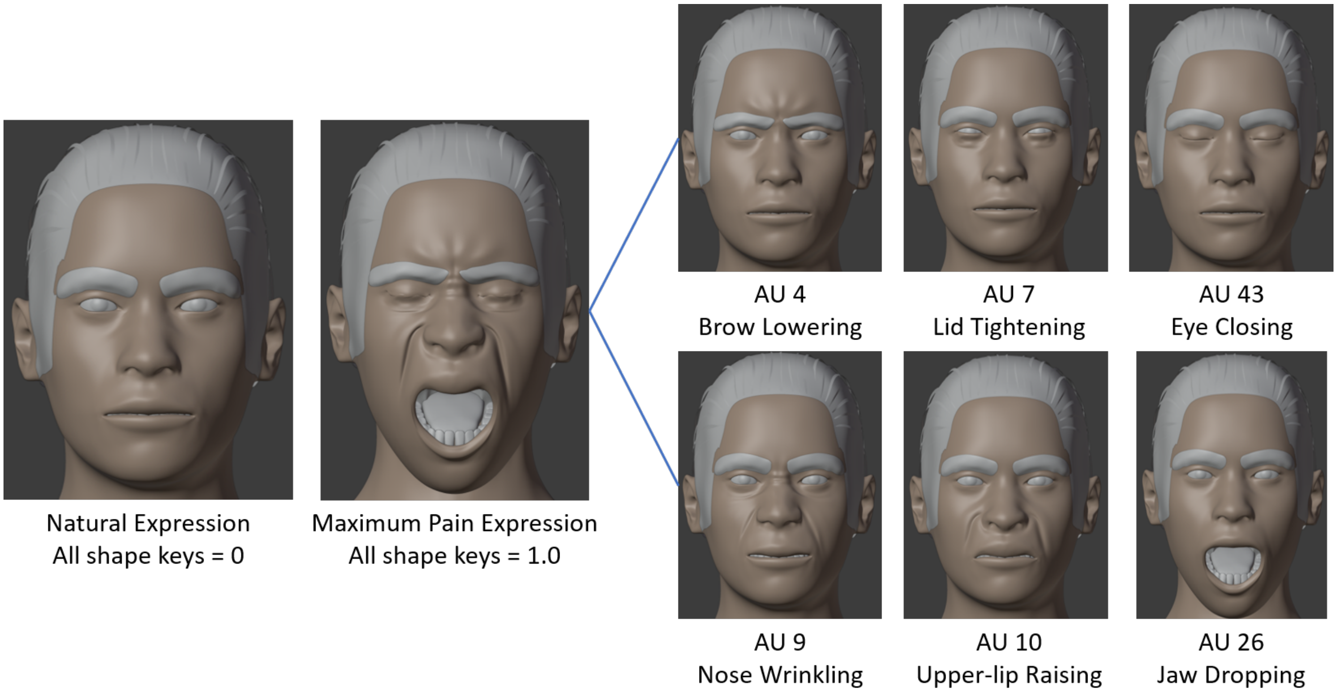

Figure S2. Solid mesh viwws of the synthesised pain facial expressions and neutral facial expressions using six pain-related AUs.

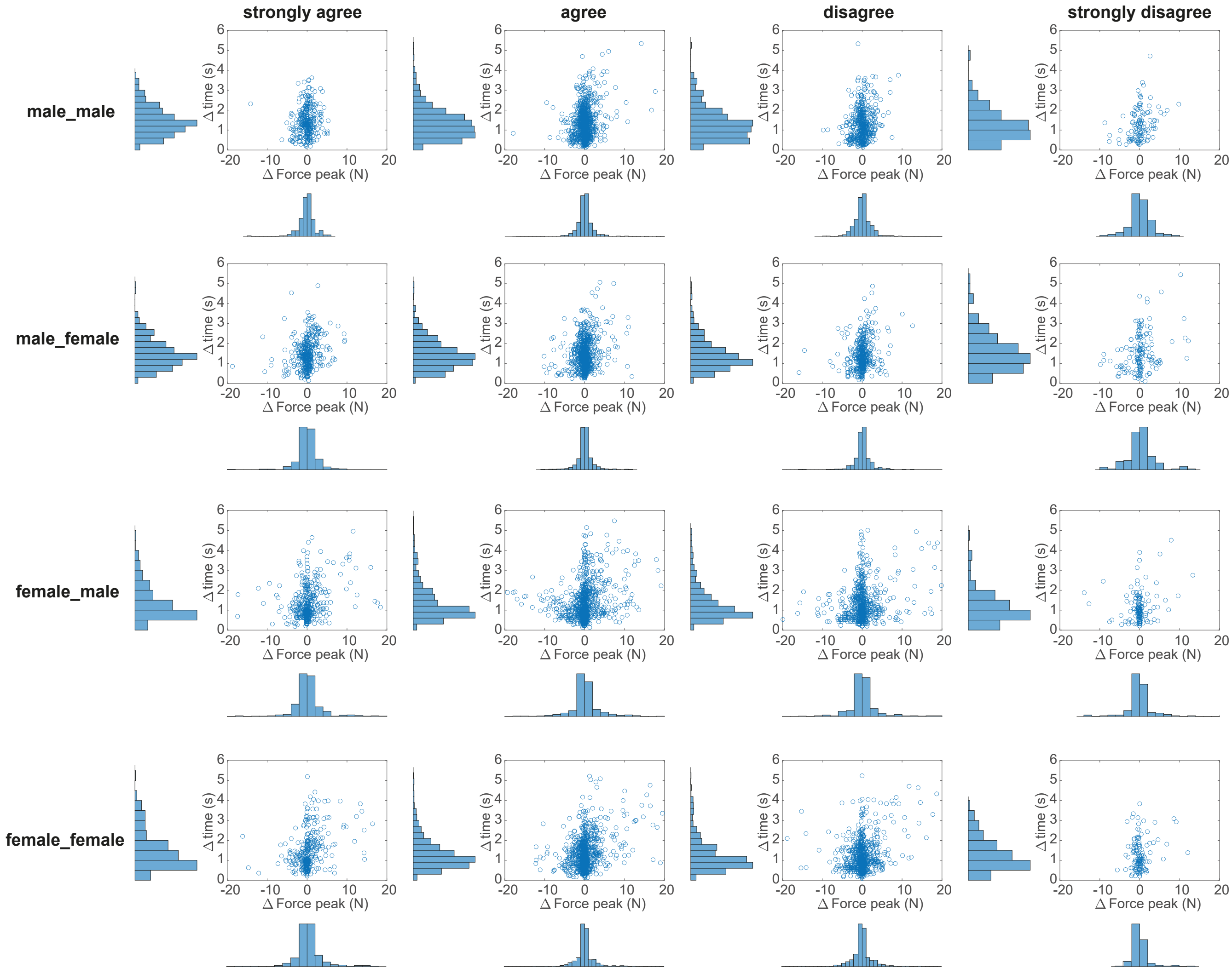

Figure S3. Distributions of the peak force differences and time differences between two consecutive palpation actions grouped by gender interactions. Row labels represent: participant gender _ Morphface gender

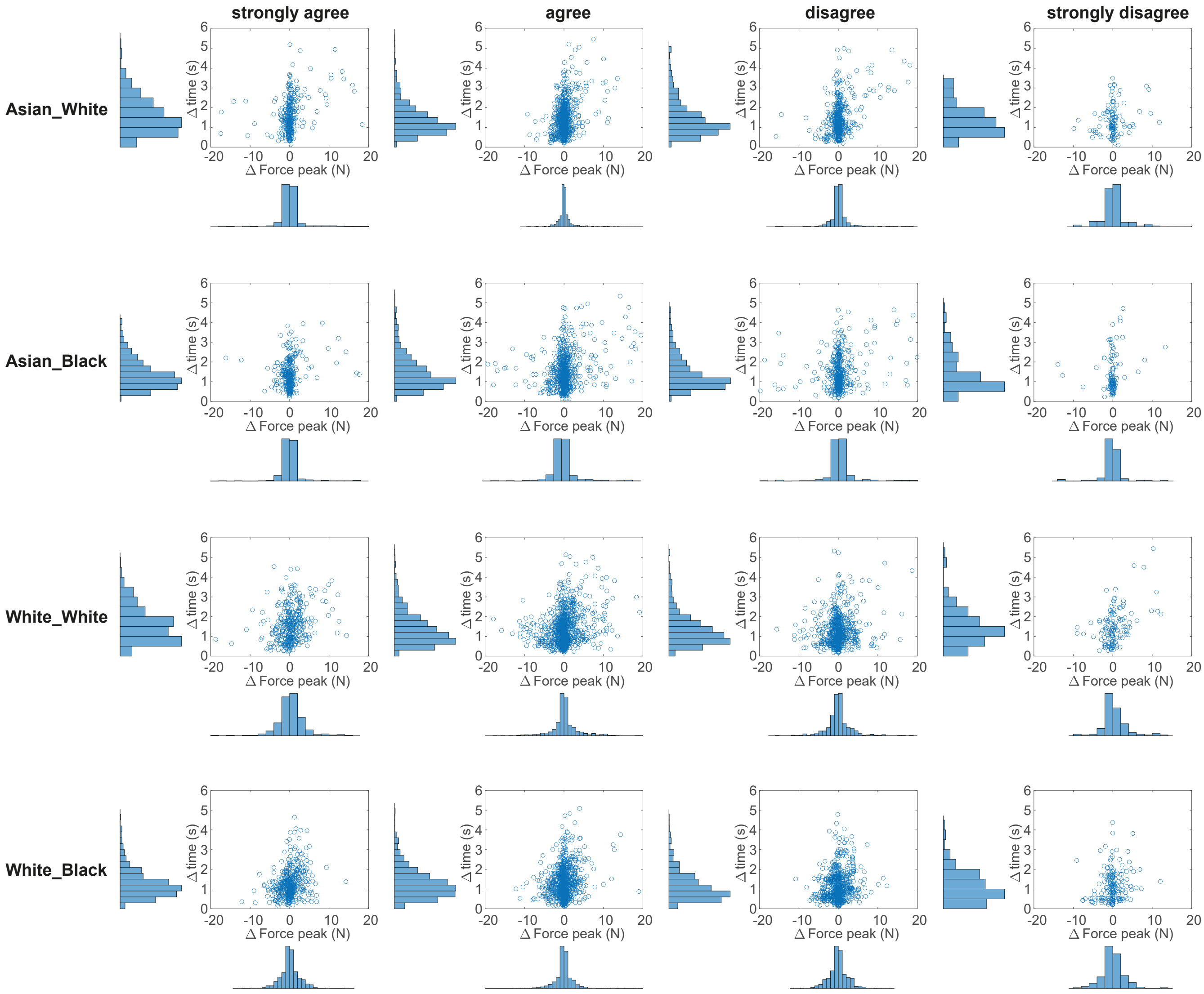

Figure S4. Distributions of the peak force differences and time differences between two consecutive palpation actions grouped by ethnicity. Row labels represent: participant ethnicity _ MorphFace ethnicity

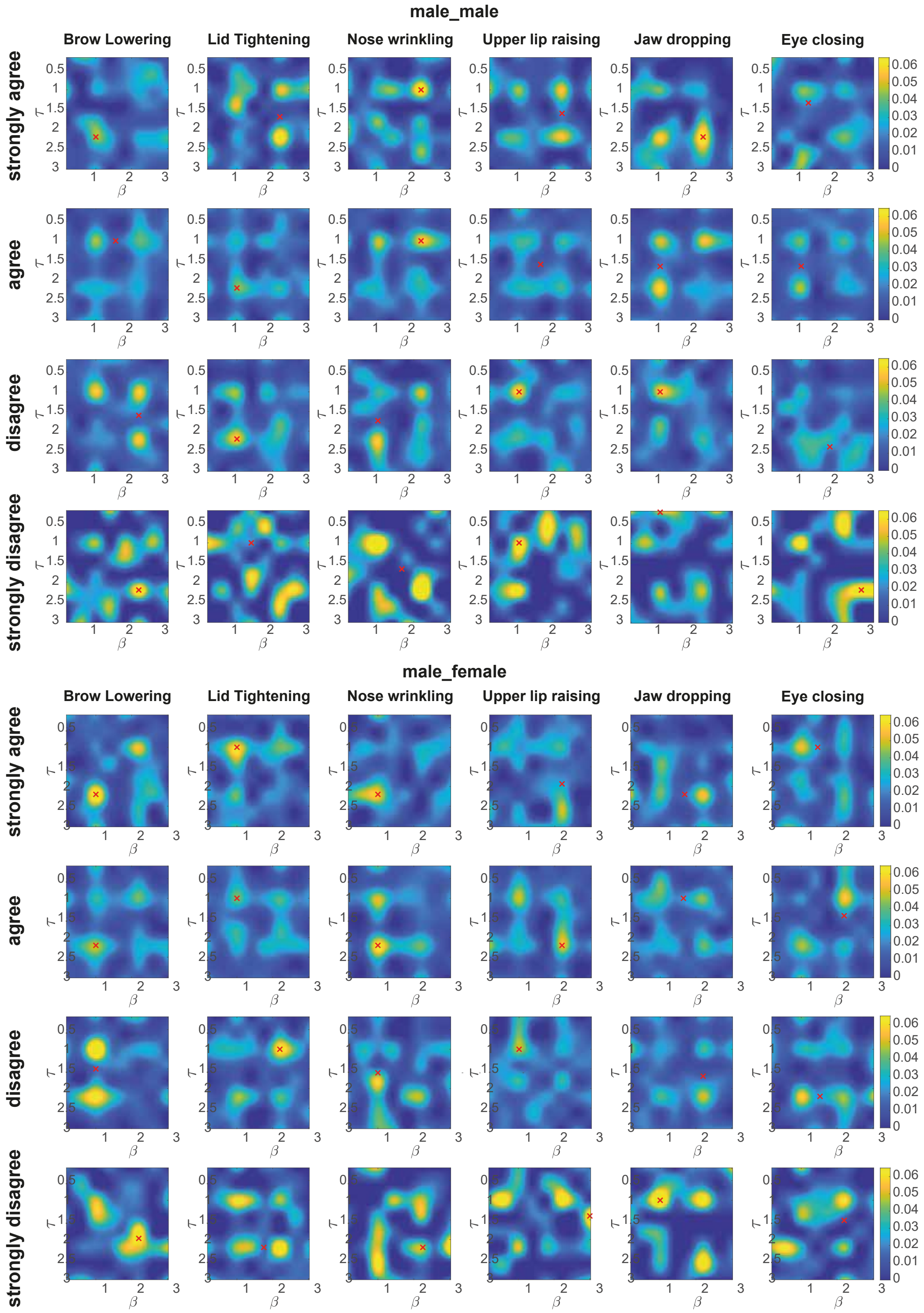

Figure S5. Probability distribution of transient parameter values for each AU across trials, grouped by gender (part 1). The weighted averages are marked by a red cross. figure labels represent: participant gender _ Morphface gender

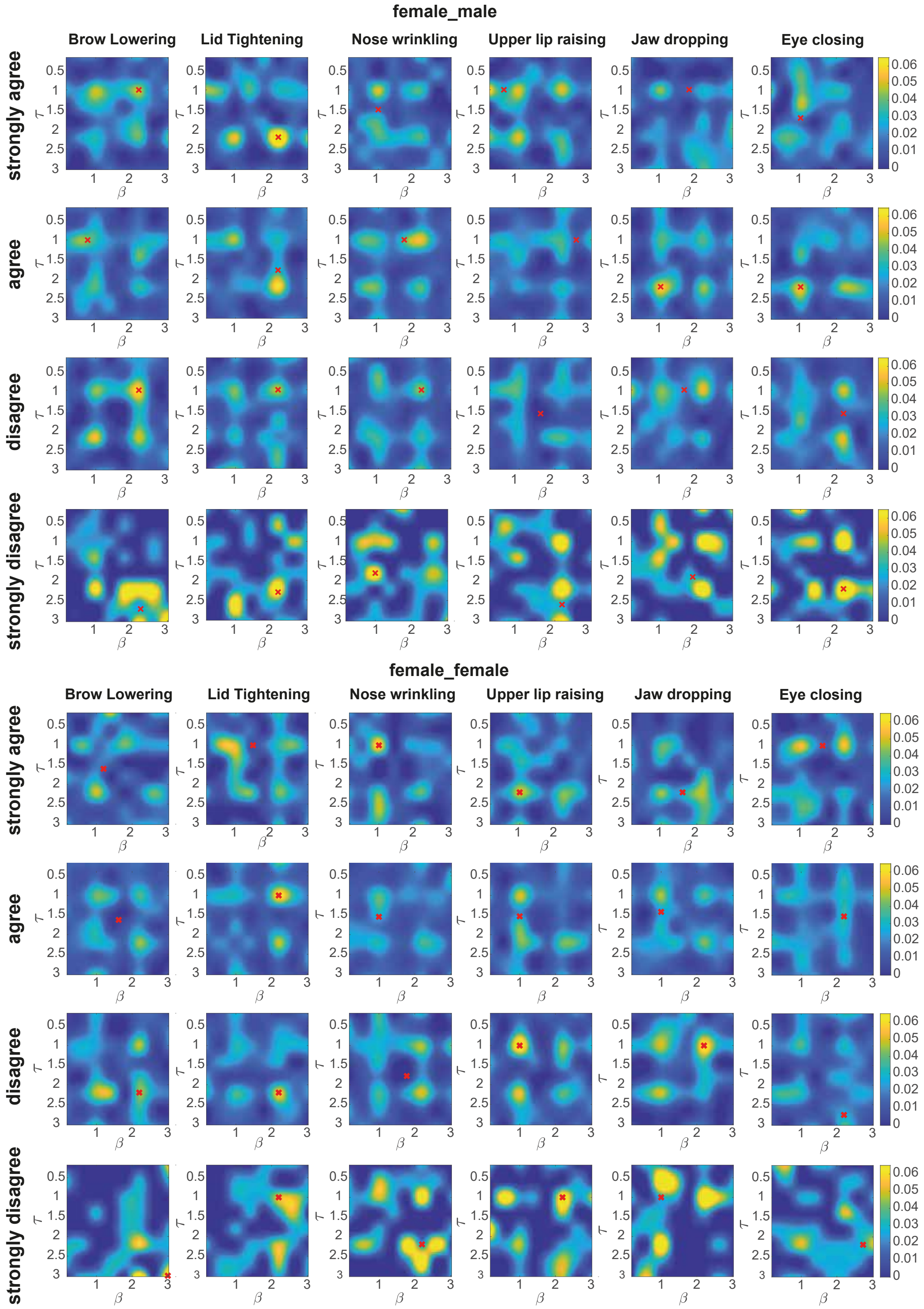

Figure S6. Probability distribution of transient parameter values for each AU across trials, grouped by gender (part 2). The weighted averages are marked by a red cross. figure labels represent: participant gender _ Morphface gender

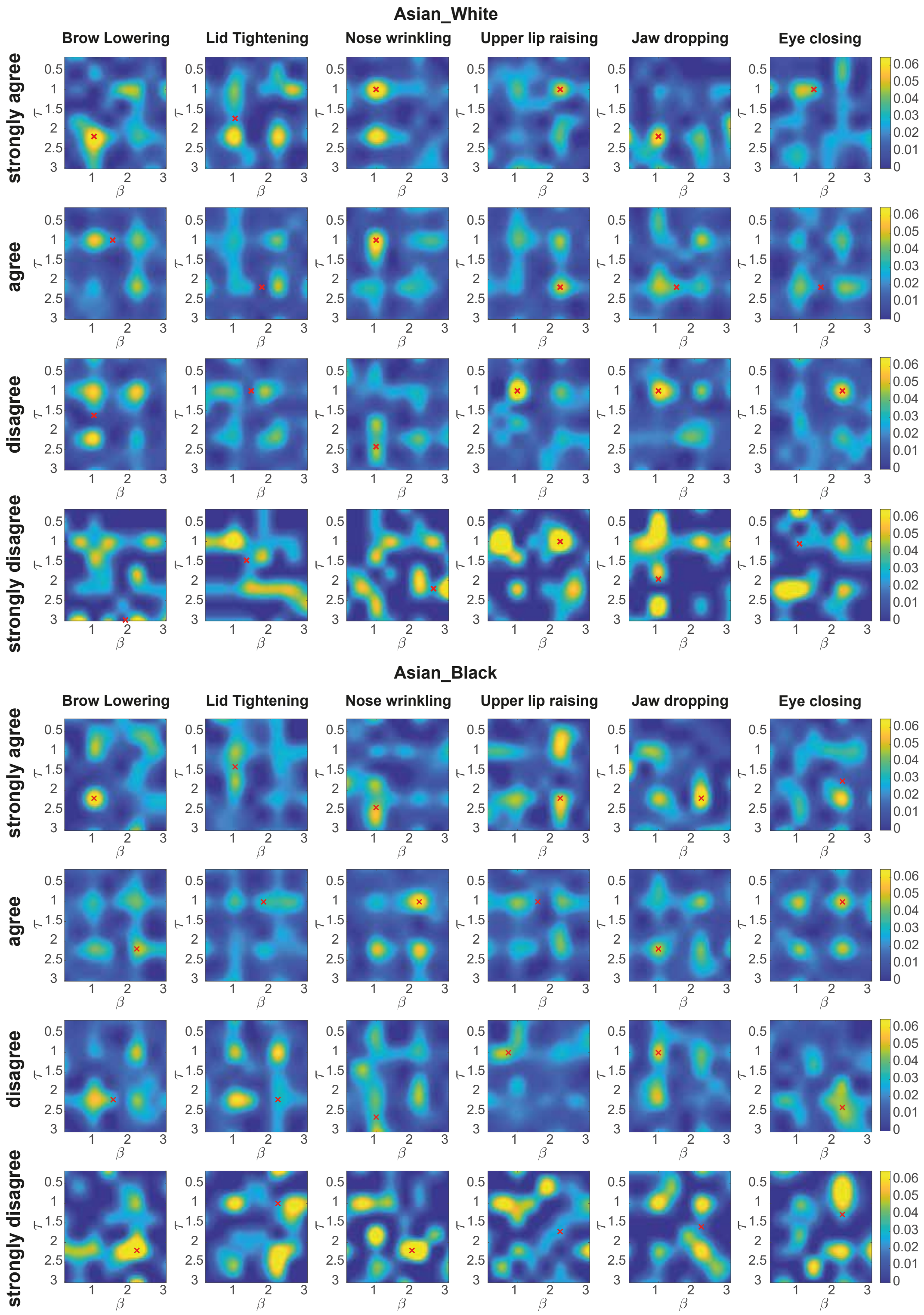

Figure S7. Probability distribution of transient parameter values for each AU across trials, grouped by ethnicity (part 1). The weighted averages are marked by a red cross. figure labels represent: participant ethnicity _ MorphFace ethnicity

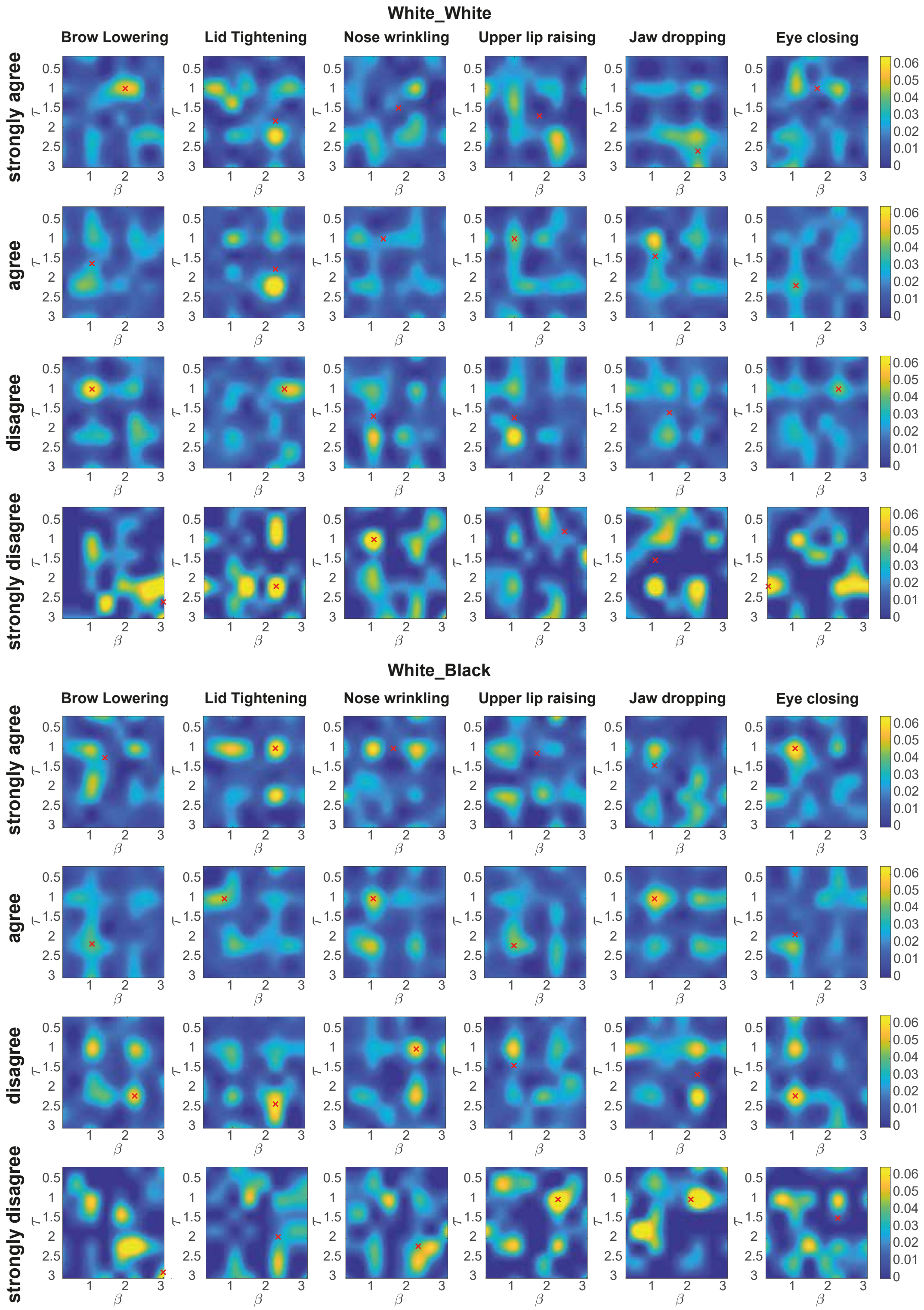

Figure S8. Probability distribution of transient parameter values for each AU across trials, grouped by ethnicity (part 2). The weighted averages are marked by a red cross. figure labels represent: participant ethnicity _ MorphFace ethnicity

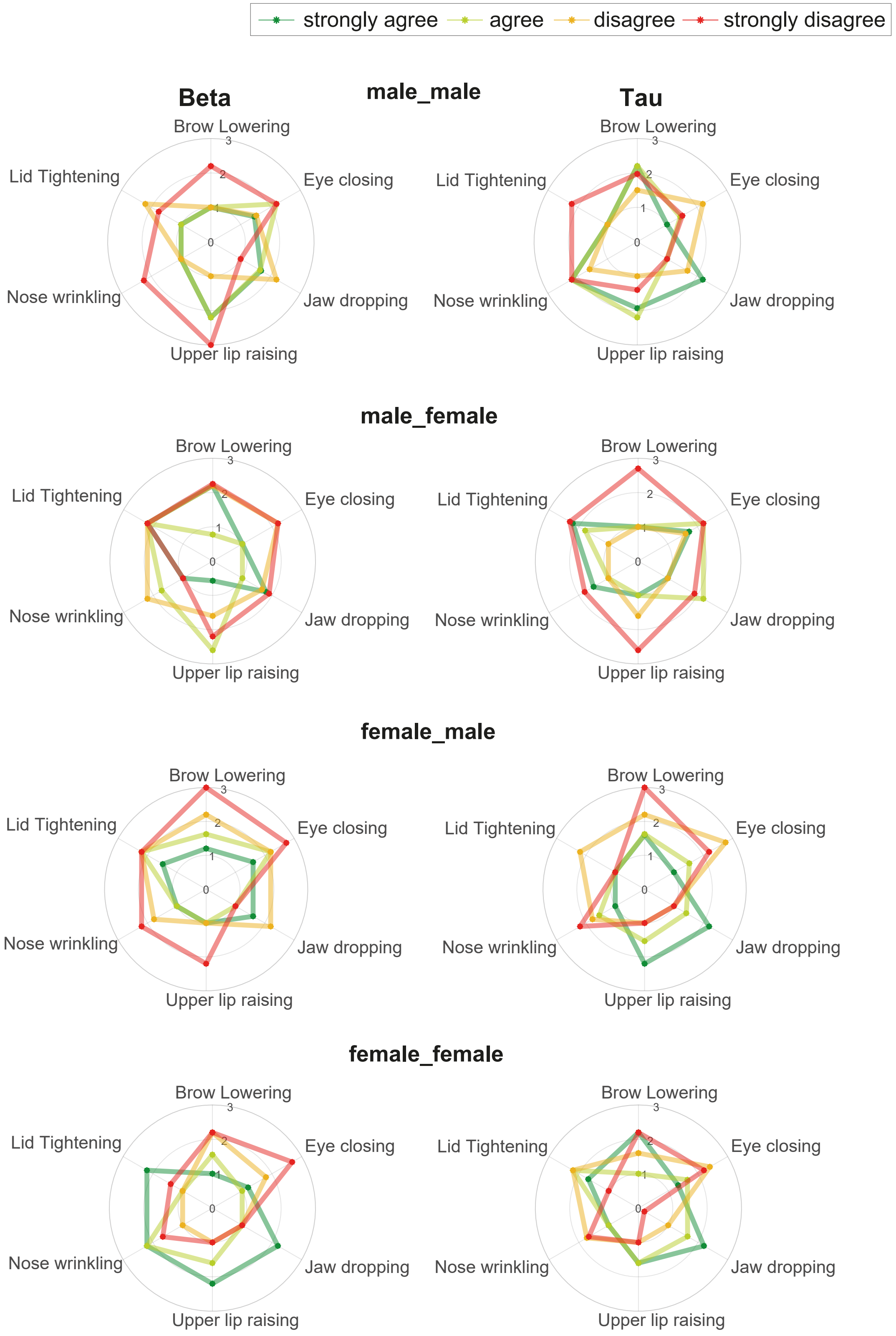

Figure S9. Average β and τ parameter variations across the 6 AUs and 4 rating cases by gender pairs.

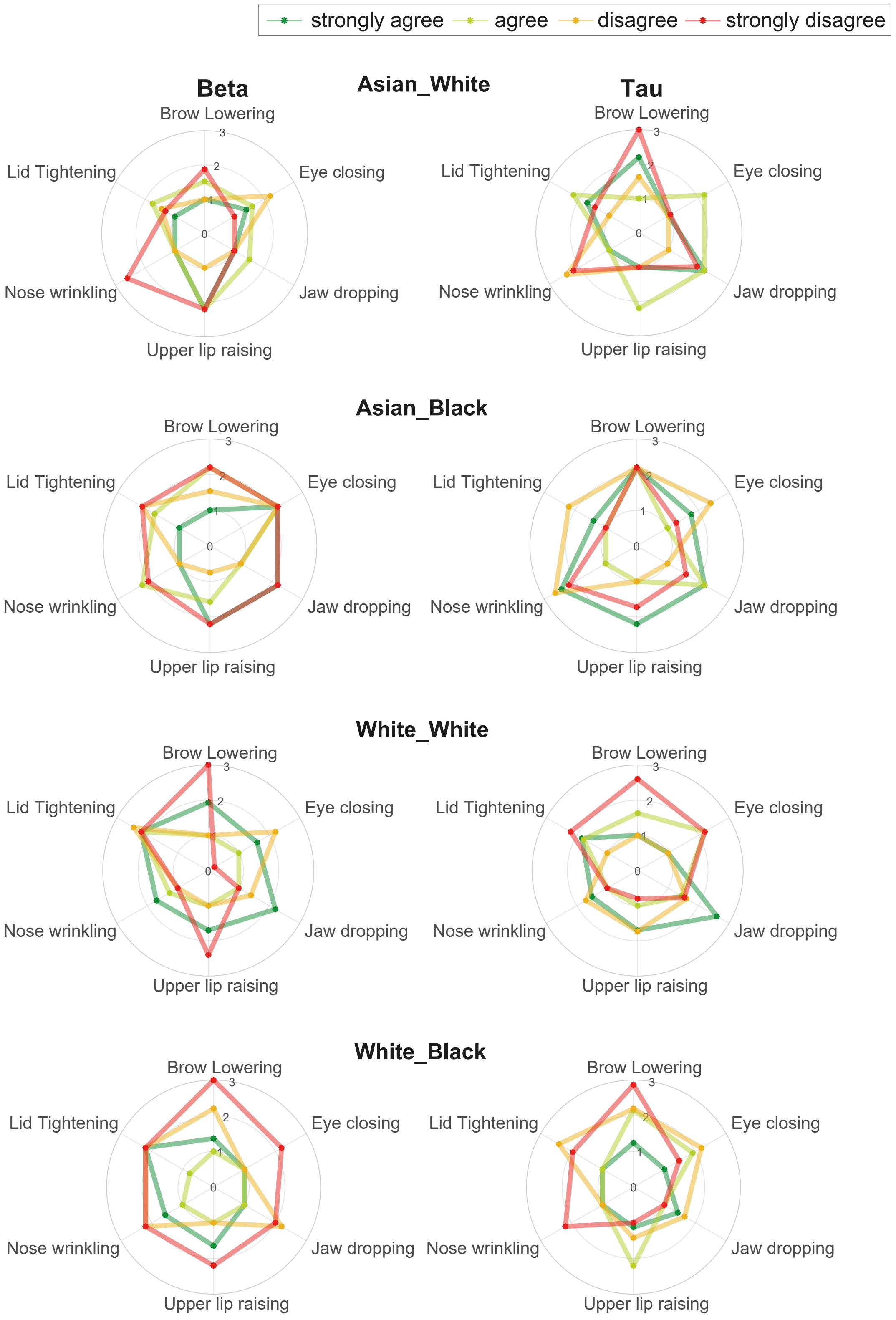

Figure S10. Average β and τ parameter variations across the 6 AUs and 4 rating cases by ethnicity pairs.
